# Phonological Task Enhances the Frequency-Following Response to Deviant Task-Irrelevant Speech Sounds

**DOI:** 10.3389/fnhum.2019.00245

**Published:** 2019-07-16

**Authors:** Kimmo Alho, Katarzyna Żarnowiec, Natàlia Gorina-Careta, Carles Escera

**Affiliations:** ^1^Department of Psychology and Logopedics, Faculty of Medicine, University of Helsinki, Helsinki, Finland; ^2^Institute of Biomedicine, Paris Descartes University, Paris, France; ^3^Brainlab-Cognitive Neuroscience Research Group, Department of Clinical Psychology and Psychobiology, Institute of Neurosciences, University of Barcelona, Barcelona, Spain; ^4^Institute of Neurosciences, University of Barcelona, Barcelona, Spain; ^5^Institut de Recerca Sant Joan de Déu, Esplugues de Llobregat, Barcelona, Spain

**Keywords:** audition, speech, electroencephalography, frequency-following response, phonological task

## Abstract

In electroencephalography (EEG) measurements, processing of periodic sounds in the ascending auditory pathway generates the frequency-following response (FFR) phase-locked to the fundamental frequency (F0) and its harmonics of a sound. We measured FFRs to the steady-state (vowel) part of syllables /ba/ and /aw/ occurring in binaural rapid streams of speech sounds as frequently repeating *standard* syllables or as infrequent (*p* = 0.2) *deviant* syllables among standard /wa/ syllables. Our aim was to study whether concurrent active phonological processing affects early processing of irrelevant speech sounds reflected by FFRs to these sounds. To this end, during syllable delivery, our healthy adult participants performed tasks involving written letters delivered on a computer screen in a rapid stream. The stream consisted of vowel letters written in red, infrequently occurring consonant letters written in the same color, and infrequently occurring vowel letters written in blue. In the *phonological task*, the participants were instructed to press a response key to the consonant letters differing phonologically but not in color from the frequently occurring red vowels, whereas in the *non-phonological task*, they were instructed to respond to the vowel letters written in blue differing only in color from the frequently occurring red vowels. We observed that the phonological task enhanced responses to deviant /ba/ syllables but not responses to deviant /aw/ syllables. This suggests that active phonological task performance may enhance processing of such small changes in irrelevant speech sounds as the 30-ms difference in the initial formant-transition time between the otherwise identical syllables /ba/ and /wa/ used in the present study.

## Introduction

Baddeley’s influential working-memory model (e.g., Baddeley and Hitch, 1974; Baddeley, 1992) proposes that the so-called articulatory-phonological loop underlies auditory working memory and is also involved in processing of written visual inputs. This model was supported by our recent functional magnetic resonance imaging (fMRI) results (Salo et al., 2013). According to our results, auditory cortex (AC) activity in response to spoken syllables is attenuated during phonological processing of written consonant letters in relation to AC activity elicited by the spoken syllables during non-phonological tasks involving the letters, that is, discriminating their font color or location rather than their phonological content. This pattern of results suggests that phonological processing of written letters occupies same phonological processing systems as processing of speech signals. In addition, our fMRI study showed enhanced AC activity during discrimination tasks involving the spoken syllables in relation to AC activity elicited by the same syllables when they were to be ignored during the visual discrimination tasks involving the written letters. Such attention-related modulation of AC activity is a common finding in fMRI studies on attention to speech or non-speech sounds (for a review and meta-analysis, see Alho et al., 2014), as well as in related studies applying electro- or magnetoencephalography (EEG and MEG, respectively; for reviews, see Näätänen et al., 2002; Fritz et al., 2007; Alain et al., 2013).

Effects of attention on auditory processing have been also found in subcortical structures of the ascending auditory pathway. For example, in their fMRI study, Rinne et al. (2008) found an effect of selective auditory attention on the activity of the inferior colliculus (IC), a brainstem nucleus in the auditory pathway from the inner ear to AC. Participants’ selective attention to a stream of tones delivered to one ear while they ignored a stream delivered to the other ear was associated with enhanced activity in the AC (see also Alho et al., 1999) and IC contralateral to the attended input in relation to activity in the ipsilateral AC and IC, respectively. This suggests attention-related facilitation of auditory processing in the AC and IC.

In EEG measurements, processing of periodic sounds in the ascending auditory pathway generates the frequency-following response (FFR) phase-locked to the fundamental frequency (F0) of a sound and its harmonics (H2, H3, etc.; e.g., Skoe and Kraus, 2010). The FFR is assumed to reflect phase-locked activity in subcortical structures of the auditory pathway including the cochlear nucleus, IC and medial geniculate body (MGB) of the thalamus, but it also gets a contribution from the AC at least for frequencies up to 120 Hz (e.g., Chandrasekaran and Kraus, 2010; Coffey et al., 2016; Bidelman, 2018).

According to several studies, attention modulates FFRs. Galbraith et al. (1998) and Lehmann and Schönwiesner (2014) found larger FFR amplitudes for a vowel delivered to one ear and attended by the listeners than for another vowel delivered simultaneously to the opposite ear, suggesting attention-related modulation of auditory processing. Moreover, Hairston et al. (2013) observed attenuated FFRs to task-irrelevant tones when participants performed duration discrimination tasks involving task-relevant auditory or visual stimuli compared with a no-task condition. Attention-related facilitation and suppression of auditory processing reflected by FFRs might be mediated by efferent connections descending from AC to the subcortical auditory nuclei, the so-called corticofugal auditory pathway (Oatman and Anderson, 1980; Galbraith et al., 1998, 2003; Suga et al., 2002; Winer, 2006; Hairston et al., 2013; Lehmann and Schönwiesner, 2014). However, as recent MEG and EEG results indicate that at least up to frequencies of 120 Hz the FFR gets also contribution from the AC (Coffey et al., 2016; Bidelman, 2018), effects of attention on FFR might be partly due to facilitation or suppression of auditory processing in the AC (see Fritz et al., 2007; Alain et al., 2013; Alho et al., 2014). This conclusion is also supported by recent results of Holmes et al. (2018) who found that attention to sounds of ca. 100 Hz may enhance FFRs to them while a similar effect was not observed for sounds of ca. 220 Hz (see also Galbraith and Kane, 1993). In contrast, Galbraith et al. (2003) found an enhancing effect of auditory attention (vs. visual attention) on FFRs elicited by tones of 293 Hz. Yet, it should be noted that many studies found no effects of direction of attention towards sounds or away from them on auditory brainstem potentials to clicks (e.g., Picton et al., 1971; Picton and Hillyard, 1974; Woods and Hillyard, 1978; Hirschhorn and Michie, 1990) or on FFRs around 100 Hz or lower (Okamoto et al., 2011; Varghese et al., 2015).

FFRs are sensitive to infrequent changes in repetitive auditory inputs. Shiga et al. (2015) measured FFRs to amplitude-modulated (AM) tones (tone duration 150 ms, carrier frequency 2,230 Hz) delivered at a constant rate of ca. 3 tones per second. Deviant tones had a higher pitch (AM frequency 410 Hz) and lower probability of occurrence (*p* = 0.2) than standard tones (AM frequency 290 Hz; *p* = 0.8) in tone streams ignored by participants watching a silent film. FFRs to deviant-pitch tones had larger amplitudes than FFRs to AM tones when they were used as standard tones in control tone streams. The pitch-deviant tones elicited also enhanced middle-latency and mismatch negativity (MMN) responses indicating change detection in the AC (for reviews, see Näätänen et al., 2007; Escera et al., 2014).

The FFR results of Shiga et al. (2015) suggesting auditory change detection already at an early processing level are supported by fMRI results of Cacciaglia et al. (2015) showing in addition to AC responses, enhanced IC and MGB responses to deviant higher-pitch noise bursts occurring among lower-pitch standard bursts delivered to participants watching a silent movie. Importantly these response enhancements were observed both in relation to brain activity elicited during stimulus blocks including only lower-pitch tones and in relation to activity elicited by noise burst varying randomly in pitch at five levels. This ruled out the possibility that the enhanced response to deviant-pitch bursts among standard-pitch bursts was simply due to the deviant bursts activating less refractory neuron populations than the standard bursts in the tonotopically organized IC, MGB, and AC.

However, brainstem processing of changes in speech sounds may differ from processing of changes in tones. Slabu et al. (2012) recorded FFRs to a syllable /ba/ replacing infrequently another syllable /wa/ in a stimulus block delivered to participants watching a silent movie with subtitles. The syllables were produced with a speech synthesizer and they differed only in the duration of transition (20 and 35 ms for /ba/ and /wa/, respectively) in their first and second formant (F1 and F2, respectively) during the initial part of the syllable. To control for effects of stimulus characteristics on FFRs, a reversed block was used where /ba/ and /wa/ swapped their status as a deviant and standard syllable. To control for simple refractoriness/adaptation effects, there was also an additional block including an infrequent /ba/ among equally infrequent four versions of /wa/ differing in their F1 and F2 transition durations. In FFRs, amplitude attenuations were observed in the second and fourth harmonics of F0 of the steady-state (vowel) portion of deviant /ba/ both in comparison to the standard /ba/, and in comparison to the infrequent /ba/ occurring among four versions of /wa/.

As reviewed above, the task performed by participants modulates FFRs (Galbraith et al., 1998; Hairston et al., 2013; Lehmann and Schönwiesner, 2014). Moreover, both speech processing and visual phonological processing have been suggested to involve the same articulatory-phonological loop (Baddeley, 1992). Therefore, the present study examined whether processing of infrequent syllable changes in the ascending auditory pathway reflected by FFRs would be affected by a concurrent phonological task involving visually presented letters.

## Materials and Methods

### Participants

Twenty-three healthy volunteers were recruited among the students of University of Barcelona. Written informed consent was obtained from all participants and they were reimbursed for collaboration with a monetary payment of 7€ per hour. The present study was approved by the Bioethics Committee of the University of Barcelona and conducted in accordance with the Code of Ethics of the World Medical Association (Declaration of Helsinki).

All participants were native speakers of Catalan or Spanish, or both. They had normal or corrected-to-normal vision, and according to their own report, no personal or familial history of psychiatric disorders, no head injuries or brain surgery, no current use of psychotropic drugs, and no hearing problems. Normal hearing of the participants was verified with a pure tone audiometry (hearing threshold at 250–8,000 Hz below 25 dB SPL for each ear). One participant was excluded from data analysis due to misunderstanding experimental task instructions and another three participants due to over 50% of their collected EEG epochs contaminated by artifacts exceeding rejection criterion (see below). The remaining 19 participants were 20–35 years old (nine males and 10 females; 11 right-handed and eight left-handed according to their own report).

### Stimuli and Procedure

Experiments were conducted in a sound-attenuated and electrically shielded room. During recordings, participants were seated comfortably in a reclining chair facing an LCD screen at 155 cm from the participant’s head. Independent sequences of written letters and spoken syllables delivered to the participants were programmed and presented using Matlab R2007a, MathWorks, and Psychophysics Toolbox Version 3 (Brainard, 1997; Pelli, 1997; Kleiner et al., 2007).

The letters (Arial font, height 1.3°–2.0°, width 1.1°–1.5°) were flashed for 50 ms in a pseudorandom order with a varying onset asynchrony of 250–500 ms (even distribution) at the center of the screen on a white (*R* = 255, *G* = 255, *B* = 255) background. Eighty percentage of letters were vowels (equiprobably A, E, O, U, a, e, o or u) written in red (*R* = 255, *G* = 0, *B* = 0), 10% of letters were vowels (equiprobably A, E, O, U, a, e, o or u) written in blue (*R* = 0, *G* = 0, *B* = 255), and 10% were consonants written in red (equiprobably B, C, D, F, G, M, N, P, R, S, T, V, Z, b, c, d, f, g, m, n, p, r, s, t, v or z). In separate blocks, the participants were instructed to respond by pressing the Enter key on the keyboard in front of them with their index or middle finger of their preferred hand either to any consonant letter or to any blue vowel. Discriminating the infrequently occurring consonants was regarded as a Phonological Task since they differed from the frequently occurring red vowels in phonology but not in color, whereas discriminating the infrequently occurring blue vowels was regarded as a Non-Phonological Task, since they differed from the frequently occurring red vowels only in a non-phonological feature, namely color. In addition, during both tasks they were instructed to ignore the stream of spoken syllables delivered in parallel with the visual stimulus stream and to keep their gaze on a black (*R* = 0, *G* = 0, *B* = 0) fixation cross (0.9° × 0.9°) visible at the center of the screen when no letter was displayed there.

The spoken syllables were generated with the Klatt speech synthesizer (Klatt, 1980) and delivered binaurally (intensity in each ear 75 dB SPL) in alternating polarities (to minimize contributions of stimulus artifact and cochlear microphonic to FFR; see, e.g., Aiken and Picton, 2008) *via* ER-3A ABR insert earphones (Etymotic Research Inc., Elk Grove Village, IL, USA) in a pseudorandom order with a varying onset asynchrony of 250–500 ms (even distribution). There were three different syllables: /ba/, /wa/, and /aw/. Each syllable had a duration of 170 ms. The syllables /ba/ and /wa/ were also used in a previous FFR study by Slabu et al. (2012). Their fundamental frequency (F0) was 100 Hz and the third (F3), fourth (F4) and fifth (F5) formants were set to 2,900, 3,500 and 4,900 Hz respectively. The first 5 ms of both /ba/ and /wa/ syllables consisted of a rapid glide in their F1 (from 400 to 1,700 Hz) and F2 (from 1,700 to 1,240 Hz), after which there was a 20-ms transition for /ba/ and 50-ms transition for /wa/ in F1 from 125 to 800 Hz and in F2 from 571 to 1,200 Hz. The syllable /aw/ was generated by presenting the syllable /wa/ backwards in time.

In Standard-/wa/ Blocks, 1,000 syllables were delivered in a pseudorandom order (i.e., each block had a duration of ca. 6 min 15 s with the visual task performed throughout the block). The syllable /wa/ was the standard syllable and occurred at a probability of 0.8, while the deviant syllables /ba/ and /aw/ at a probability of 0.1 each. Five Standard-/wa/ Blocks were delivered during the Phonological Task and another five during the Non-Phonological Task. Thus, both deviant /ba/ and deviant /aw/ occurred 500 times in each task condition.

In addition, we presented for each task condition one Standard-/ba/ Block where /ba/ was the standard syllable (*p* = 0.8) and /wa/ and /aw/ were the deviant syllables (*p* = 0.1 for each) and one Standard-/aw/ Block where /aw/ was the standard syllable (*p* = 0.8) and and /wa/ and /ba/ were the deviant syllables (*p* = 0.1 for each). This allowed us to compare FFRs to the deviants /ba/ and /aw/ in the Standard-/wa/ blocks with those to the standard /ba/ and standard /aw/ to control for effects of specific stimulus characteristics on FFRs to the deviant syllables /ba/ and /aw/. In Standard-/ba/ and Standard-/aw/ Blocks, there were 640 syllables in each (i.e., each block had a duration of ca. 4 min) including 512 standard syllables and 64 deviant syllables of each type. Note that the deviant syllables were delivered in Standard-/aw/ and Standard-/ba/ Blocks just to keep the stimulus probability structure within these blocks analogous to that in the Standard-/wa/ Blocks. Due to the small number of deviant syllables in these blocks, FFRs to these deviant syllables were not analyzed.

Thus, altogether 14 blocks were delivered to each participant and the duration of the experiment, including short 1–2 min breaks between the blocks, was about 1 h 30 min. The order of blocks and task conditions was randomized separately for each participant.

### EEG Data Acquisition, Processing and Analysis

To obtain FFRs, EEG (0.05–3,000 Hz; sampling rate 20 kHz) was recorded during the experimental blocks with SynAmps RT amplifier (Compumedics Neuroscan, Charlotte, NC, USA) and Neuroscan 4.4 acquisition software as a voltage between the fronto-central midline (FCz) Ag/AgCl scalp electrode in the Neuroscan Quik-Cap electrode system and an Ag/AgCl electrode attached to the right earlobe (A2). The default Quick-Cap ground electrode was located between the frontal (Fz) and fronto-polar (Fpz) midline sites. All electrode impedances were kept below 5 kΩ.

Analysis of EEG data was performed using Matlab R2012a, MathWorks, and EEGLAB, an open source toolbox for analysis of single-trial EEG dynamics (Delorme and Makeig, 2004). First, frequencies between 70 Hz and 1,500 Hz were filtered from the EEG data with a Kaiser finite impulse response (FIR) bandpass filter (transition bandwidth 15 Hz, passband ripple 0.001). Then FFRs were obtained for the deviant /ba/ and /aw/ syllables of the Standard-/wa/ blocks and for the standard /ba/ and /aw/ syllables of the Standard-/ba/ and Standard-/aw/ blocks, respectively, separately for each participant and separately for Phonological and Non-Phonological Tasks by averaging EEG epochs starting 40 ms before each syllable onset and ending 180 ms after syllable onset. The 0-μV baseline was set at the mean amplitude during the 40-ms pre-syllable period. Epochs with voltages exceeding ±35 μV were rejected from averaging. As noted above, deviants /wa/ and /aw/ in Standard-/ba/ blocks and deviants /ba/ and /wa/ in Standard-/aw/ blocks were excluded from data analysis due to their small number. Consequently, data for the standard /wa/ were not analyzed as there were not enough data for the deviant /wa/ with which data for the standard /wa/ could be compared.

To analyze FFR amplitudes in the frequency domain during the vowel (steady-state) part of syllables, fast Fourier transform was applied on each participant’s demeaned, zero-padded (1-Hz resolution) and Hanning-tapered FFRs. A time window 35–165 ms from syllable onset was used for the FFRs to the deviant and standard /ba/, because /ba/ started with formant transitions due to the initial consonant and ended in a vowel with steady-state formants. For the deviant and standard /aw/, in turn, a time window 10–115 ms from syllable onset was used in the FFR analysis, because /aw/ started with steady-state formants and ended in formant transitions (Note that the FFRs to the syllables /ba/ and /aw/ were not compared statistically with each other and therefore the different time windows used to obtain these FFRs did not affect the statistical results.). The mean FFR amplitude was computed separately for each participant, and separately for the deviant /ba/, standard /ba/, deviant /aw/, and standard /aw/ during Phonological and Non-Phonological Tasks using 10-Hz wide windows centered at the fundamental frequency (F0) of syllables at 100 Hz and at the second (H2) and third harmonic (H3) at 200 and 300 Hz, respectively.

In addition to FFRs, we analyzed long-latency ERPs to deviant and standard syllables in order to study MMN responses elicited by deviant /ba/ and /aw/ syllables. For this analysis, the EEG recorded at the FCz electrode site was resampled at 500 Hz and filtered using a Kaiser FIR filter with a passband of 0.5–20 Hz. ERPs were obtained by averaging EEG epochs starting 40 ms before syllable onset and ending 400 ms after syllable onset. This was done separately for the deviant /ba/ and /aw/ syllables of the Standard-/wa/ blocks and for the standard /ba/ and /aw/ syllables of the Standard-/ba/ and Standard-/aw/ blocks, respectively, separately for each participant, and separately for Phonological and Non-Phonological Tasks. The 0-μV baseline for amplitude measurements was set at the mean amplitude during the 40-ms pre-syllable period. Epochs with voltages exceeding ±35 μV were rejected from averaging.

### Statistical Analysis

Button presses given 100–1,100 ms after target-letter onset were regarded as hits. Other responses were classified as false alarms. Hit response times, hit rates (number of hits divided by the number of target letters) and false-alarm rates (number of false alarms divided by the number of non-target letters) were calculated for each participant across the blocks of separately for Phonological and Non-Phonological Tasks and then subjected to one-way repeated-measures analysis of variance (ANOVAs) in order to compare participants’ performance speed and accuracy in these tasks. Effects with *p* ≤ 0.05 were regarded as significant and effect sizes ($\eta_{\text{p}}^{2}$) were calculated for these cases. It should be noted that since the stimulus onset asynchrony for the letters was randomly 250–500 ms, there is a risk that a delayed hit response to a target letter was classified as a false alarm to a subsequent non-target letter or that false-alarm responses to non-targets given during the 100–1,000 ms time windows following targets were classified as hits. However, probabilities for such misclassifications of responses were similar in Phonological and Non-Phonological Visual Tasks. Therefore, the estimated hit and false-alarm rates are still comparable between these tasks.

Peak amplitudes of frequency spectra of FFRs (measured as mean amplitudes in 10-Hz windows centered at the peak frequency) were analyzed with two-way repeated-measures ANOVAs, performed separately for the FFRs to syllables /ba/ and /aw/, and including factors Task (Phonological vs. Non-Phonological) and Deviance (deviant vs. standard syllable). In case an ANOVA showed significant (*p* ≤ 0.05) effects of factors or their significant interaction, effect sizes ($\eta_{\text{p}}^{2}$) were calculated and subsequent *post hoc t*-tests (with Bonferroni-corrected significance criterion of *p* ≤ 0.0125) were performed for within-condition (Phonological or Non-Phonological Task) comparisons between FFRs to standard and deviant syllables and between-condition comparisons separately for standard syllables and deviant syllables if an ANOVA showed significant effects.

To evaluate significance of differences between long-latency ERPs to deviant and standard /ba/ syllables and between long-latency ERPs to deviant and standard /aw/ syllables due to MMN responses elicited by the deviant syllables, the mean amplitudes of these ERPs during Phonological and Non-Phonological Tasks were measured separately for each participant over consecutive 100-ms periods from syllable onset, that is, over 0–100, 100–200, 200–300, and 300–400 ms. Statistical significance of effects of the factors Deviance (deviant vs. standard syllable) and Task (Phonological vs. Non-Phonological) and their interaction was assessed with a repeated-measures ANOVA performed separately for ERPs to syllables /ba/ and /aw/. Since four dependent ANOVAs were performed for ERPs to each syllable, instead of using a significance criterion of *p* ≤ 0.05, Bonferroni-corrected criterion of *p* ≤ 0.0125 was applied and effect sizes ($\eta_{\text{p}}^{2}$) were calculated only for effects fulfilling this corrected criterion.

## Results

### Task Performance

**Figure 1:**
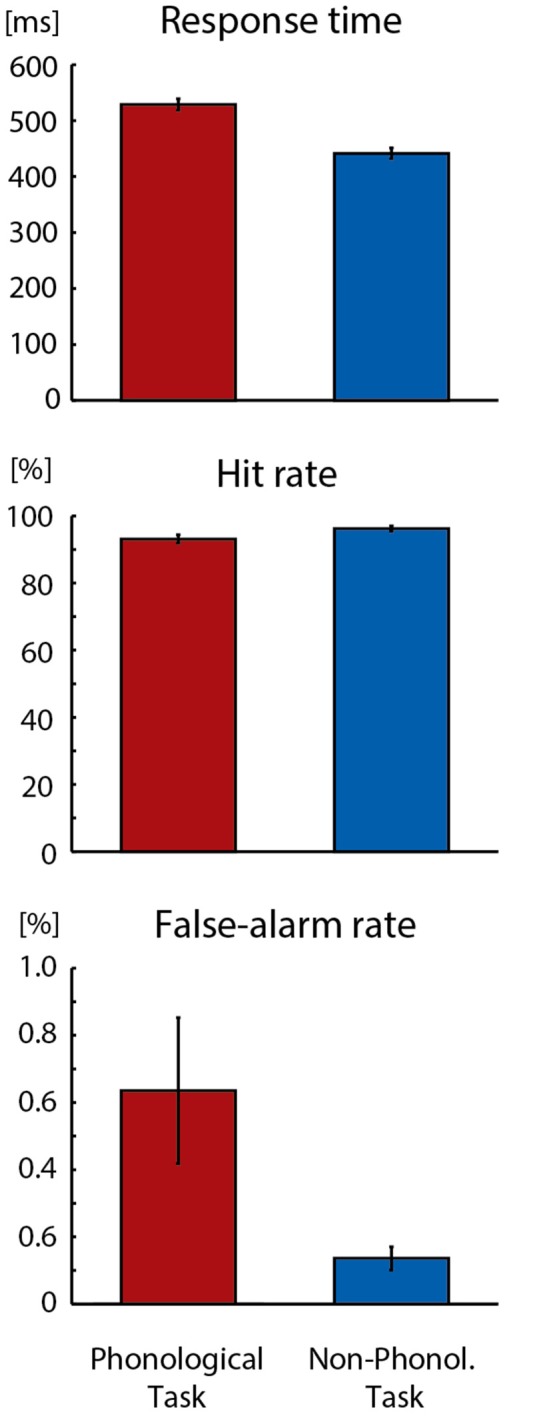
Mean response times, hit rates and false-alarm rates in detecting visual target stimuli in Phonological and Non-Phonological Tasks. Error bars indicate standard errors of the mean.

As could be expected, the Phonological Task was somewhat more difficult than the Non-Phonological Task. As seen in Figure 1, the participants’ response times to target letters were longer in the Phonological Task than in the Non-Phonological Task (*F*_(1,18)_ = 246.457, *p* < 0.001, $\eta_{\text{p}}^{2}$ = 0.93). Moreover, their hit rates in detecting target letters were lower in the Phonological than Non-Phonological Task (*F*_(1,18)_ = 15.108, *p* < 0.002, $\eta_{\text{p}}^{2}$ = 0.46) and they made more false alarms in Phonological Task (*F*_(1,18)_ = 5.676, *p* < 0.03, $\eta_{\text{p}}^{2}$ = 0.24).

### FFRs

**Figure 2:**
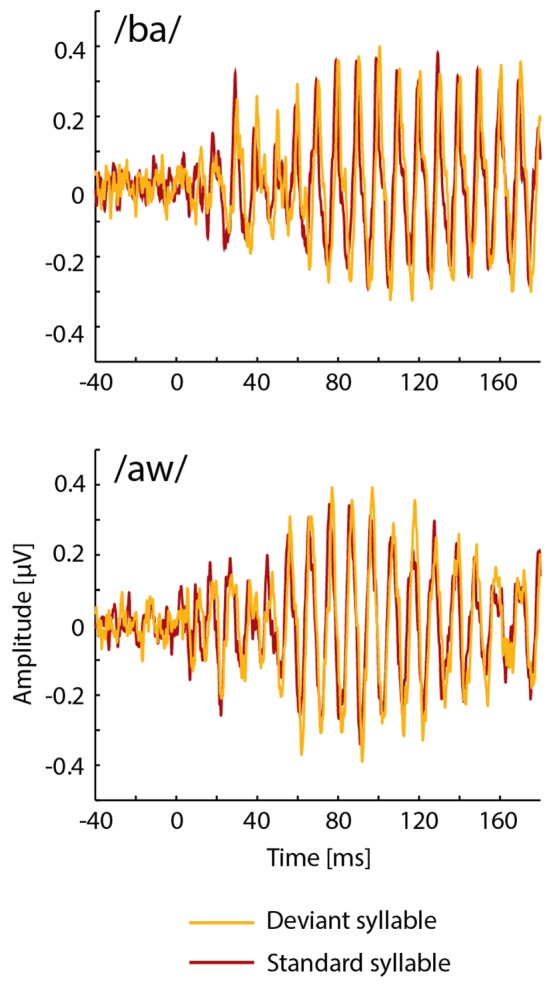
Across 19 participants averaged frequency-following responses (FFRs) to deviant (orange lines) and standard (red lines) syllables /ba/ (top panel) and /aw/ (bottom panel) delivered binaurally *via* earphones during the visual Phonological Task involving letters displayed on a computer screen.

**Figure 3:**
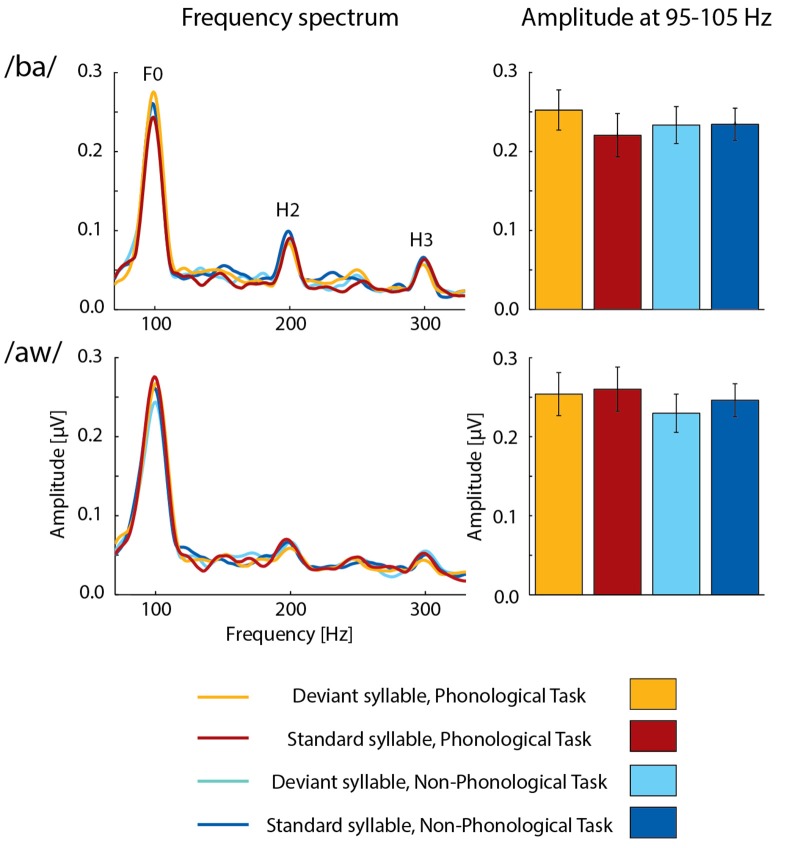
Left column: across 19 participants averaged frequency spectra of FFRs to deviant and standard /ba/ syllables (top row) and deviant and standard /aw/ syllables (bottom row) during the visual Phonological Task (deviants: orange lines, standards: red lines) and Non-Phonological Task (deviants: cyan lines, standards: blue lines). The labels F0, H2, and H3 indicate peaks of fundamental frequency and its second and third harmonics, respectively, in the frequency spectra. Right column: the mean F0 amplitudes (average amplitudes over 95–105 Hz) measured from the frequency spectra for deviant and standard /ba/ and /aw/ syllables during the visual Phonological Task (deviants: orange bars, standards: red bars) and Non-Phonological Task (deviants: cyan bars, standards: blue bars). Error bars indicate standard errors of the mean.

FFRs averaged for deviant and standard /ba/ and /aw/ syllables are shown in Figures 2, 3 depict frequency spectra of these FFRs and mean amplitudes in these around F0 during Phonological and Non-Phonological Tasks.

A two-way ANOVA for FFR amplitudes over 95–105 Hz (i.e., around the F0 frequency of 100 Hz) to syllable /ba/ indicated a significant effect of Deviance (*F*_(1,18)_ = 6.158, *p* < 0.025, $\eta_{\text{p}}^{2}$ = 0.25), but no significant effect of Task. However, there was a significant Task × Deviance interaction (*F*_(1,18)_ = 4.733, *p* < 0.05, $\eta_{\text{p}}^{2}$ = 0.21). As seen in Figure 3, FFRs were larger to deviant /ba/ than to standard /ba/ during Phonological Task but not during Non-Phonological task. Four *post hoc t*-tests (with a Bonferroni-corrected significance criterion: *p* ≤ 0.0125) comparing FFR amplitudes to deviants vs. standards within each task and to deviants or standards across the tasks indicated that during Phonological Task, the FFR amplitude around F0 to deviant /ba/ was significantly larger than that to standard /ba/ (*t*_(18)_ = 4.802, *p* < 0.0002) the other differences in these pairwise comparisons being insignificant (*p* > 0.25 in all cases) and the insignificant difference between deviant and standard /ba/ during Non-Phonological Task actually being of opposite polarity (see Figure 3). Thus, the significant Task × Deviance interaction indeed resulted from enhanced deviant vs. standard FFR difference during Phonological Task in relation to Non-Phonological Task, but it is not possible to judge from the present data whether attenuation of FFR to standard /ba/ (see Figure 3) during Phonological Task in relation to Non-Phonological Task also contributed to this deviant vs. standard FFR difference.

A two-way ANOVA for F0 amplitudes to deviant /aw/ and standard /aw/, in turn, showed no significant effects of Deviance or Task, or significant Task × Deviance interaction. Furthermore, ANOVAs for the amplitudes of H2 and H3 harmonics in FFRs (see Figure 3, left column) showed no significant effects of Task or Deviance or significant Task × Deviance interaction for either syllable /ba/ or /aw/.

As seen in Figure 2, unexpectedly, the FFRs to deviant syllables /ba/ and /aw/ appeared to be slightly delayed in relation to the FFRs to the respective standard syllables. In order to analyze this in detail, cross correlations were calculated between FFRs to deviant and standard /ba/ and between FFRs to deviant and standard /aw/ (see Russo et al., 2004; Ribas-Prats et al., 2019) separately for Phonological and Non-Phonological Tasks and separately for each participant. According to these cross-correlation analyses, the FFRs to deviant syllables tended to lag in relation to FFRs to standard syllables, this lag being on average 1.7 ms (standard error of the mean ±3.4 ms) and 2.9 ms (±2.0 ms) for the syllable /ba/ during Phonological and Non-Phonological Tasks, respectively, and 0.9 ms (±3.3 ms) and 0.9 ms (±5.4 ms) for the syllable /aw/ during Phonological and Non-Phonological Tasks, respectively. However, subsequent *t*-tests showed that none of these lags differed significantly from 0 ms (in all four cases *t*_(18)_ < 1.42, *p* > 0.17).

### MMN

**Figure 4:**
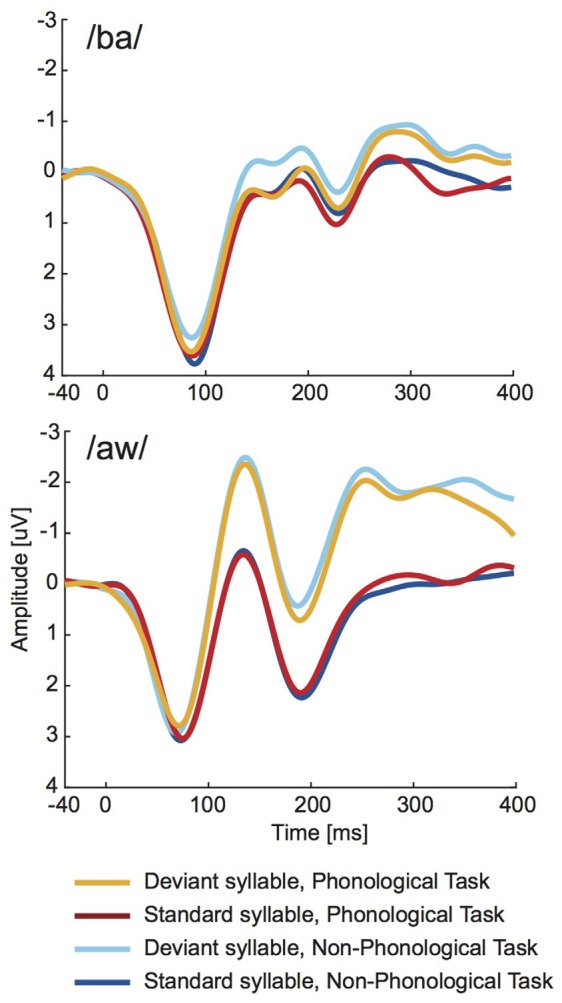
Across 19 participants averaged long-latency ERPs to deviant (orange lines) and standard (red lines) syllables /ba/ (top panel) and /aw/ (bottom panel) delivered during the visual Phonological Task and to respective deviant (cyan lines) and standard (blue lines) syllables during the visual Non-Phonological Task.

As seen in Figure 4, the long-latency ERPs to deviant syllables were negatively displaced in relation to standard syllables. Statistical significance of this difference was evaluated separately for syllable /ba/ and syllable /aw/ at four consecutive 100-ms time windows from syllable onset with two-way ANOVAs (Bonferroni corrected significance criterion: 0.0125) including factors Deviance (deviant vs. standard syllable) and Task (Phonological vs. Non-Phonological). According to these ANOVAs the mean amplitudes over 200–300 ms and over 300–400 ms from syllable onset were significantly more negative in ERPs to deviant /ba/ than in ERPs to standard /ba/ (significant effect of Deviance, 200–300 ms: (*F*_(1,18)_ = 12.945, *p* < 0.003, $\eta_{\text{p}}^{2}$ = 0.42; 300–400 ms: (*F*_(1,18)_ = 19.283, *p* < 0.001, $\eta_{\text{p}}^{2}$ = 0.52). For the /aw/ syllable, the ERPs to deviant /aw/ had significantly more negative amplitudes than ERPs to standard /aw/ at 100–200 ms (*F*_(1,18)_ = 69.107, *p* < 0.001, $\eta_{\text{p}}^{2}$ = 0.79), 200–300 ms (*F*_(1,18)_ = 33.349, *p* < 0.001, $\eta_{\text{p}}^{2}$ = 0.65), and 300–400 ms (*F*_(1,18)_ = 12.374, *p* < 0.003, $\eta_{\text{p}}^{2}$ = 0.41). None of these ANOVAs showed significant effects of Task. Neither were there significant Task × Deviance interactions, although Figure 4 suggests that the differences between ERPs to deviant and standard syllables tended to be slightly smaller during Phonological than Non-Phonological Task.

## Discussion

The aim of the present study was to clarify whether processing of infrequent syllable changes in the ascending auditory pathway reflected by FFRs would be affected by a concurrent visual phonological task. While our previous fMRI results suggested suppression of processing of to-be-ignored spoken syllables during a visual phonological task (Salo et al., 2013), the present results showed an enhancement of FFR to a phonetic change in a spoken syllable during the phonological task involving visually presented letters. This unexpected effect was observed for infrequent changes of a repeating syllable from /wa/ to /ba/. Importantly, this effect for deviant /ba/ syllables was revealed by a comparison of FFRs to deviant /ba/ syllables with FFRs to identical standard /ba/ syllables (delivered in blocks with inverted probabilities of /ba/ and /wa/ syllables) controlling for simple effects of physical stimulus features on FFR.

While the present FFR data suggest enhanced processing of deviant /ba/ syllables during the visual phonological task in comparison with the visual non-phonological task, the nature of visual task was not observed to have any significant effects on the long-latency ERPs to deviant or standard /ba/ or /aw/ syllables. However, these ERPs showed significant effects of syllable deviance: ERPs to deviant syllables were negatively displaced in relation to ERPs to standard syllables, these effects presumably being due to the MMN response elicited by deviant syllables (for reviews, see Näätänen et al., 2007; Escera et al., 2014). Since the MMN has its major generators bilaterally in the AC, the lack of effect of visual phonological task on MMN might be regarded as suggesting functional independence of MMN generator processes from the deviance detection reflected by the present FFRs to deviant /ba/ syllables and enhanced by the visual phonological task. The lack of effect of visual phonological task on the MMN supports the proposal that auditory change detection reflected by the MMN is largely independent of attention (see, e.g., Näätänen et al., 2007). This lack also rules out the possibility that the FFR enhancement observed for the deviant /ba/ syllables during the visual phonological task was due to an effect of this task on the MMN overlapping in time with the late part of FFR to deviant /ba/ syllable and therefore potentially contaminating the FFR results.

Taken the subcortical and cortical generator sources of FRRs (Coffey et al., 2016; Bidelman, 2018), the enhancement of FFRs to deviant /ba/ syllables during visual phonological processing is presumably caused by top-down modulation of speech processing in the AC or hierarchically lower structures of the auditory pathway. Since the processing of deviant /ba/ syllables in the AC reflected by the MMN elicited by these syllables was not affected by the nature of the visual task, it is more likely that the present FFR enhancement observed for the deviant /ba/ syllables during the visual phonological task originated from subcortical structures of the ascending auditory pathway rather than from the AC. However, this enhancement might be due to top-down modulation of activity in these subcortical structures *via* corticofugal connections descending from the AC (see Suga et al., 2002; Galbraith et al., 2003; Winer, 2006).

No difference was observed in the FFRs to deviant /aw/ syllables between the visual phonological and non-phonological tasks. This suggests that the visual phonological task facilitates especially processing of small contrasts between speech sounds, like the 30-ms difference in the frequency transition time between the present /ba/ and /wa/ syllables, rather than processing of large differences, like the difference between the present /aw/ and /wa/ syllables starting without and with a frequency transition, respectively. Yet, it should be borne in mind that all syllables used in the present study contained the same frequencies and therefore it remains to be studied whether FFRs to infrequent small or large changes in the pitch (F0) or frequency structure of spoken syllables or other sounds (see Cacciaglia et al., 2015; Shiga et al., 2015) would be affected by a concurrent phonological task. However, it should be noted that the present /aw/ vs. /wa/ contrast was created by presenting the same syllable /wa/, perceived as a diphthong, forwards or backwards. While such temporal deviancies have been shown to elicit cortical MMN responses (Sams and Näätänen, 1991; Pardo and Sams, 1993), to our knowledge, no previous study has shown that processing of such higher-order stimulus contrasts would be reflected by FFRs.

In their study, Hairston et al. (2013) demonstrated attenuation of FFRs to task-irrelevant sounds during attention-demanding tasks involving other sounds or visual stimuli in comparison with a no-task baseline. Since the present study did not include a no-task condition it is not possible to estimate whether such suppression occurred during the present visual tasks. However, if this suppression were related to attention, one would have expected to see more attenuated FFRs to both standard and deviant syllables during the phonological visual task than during the non-phonological visual task. This is because the present visual phonological task was, according to the present performance speed and accuracy results, more difficult, and thus presumably more attention-demanding, than the visual non-phonological tasks.

In the present study, we observed enhanced amplitudes at the F0 frequency of the FFR in response to deviant /ba/ syllables occurring among standard /wa/ syllables during the visual phonological task in relation to standard /ba/ syllables delivered during a similar visual phonological task. In contrast, Slabu et al. (2012) observed attenuated FFR amplitudes at the second and fourth harmonics of F0 in response to deviant /ba/ syllables in relation to the standard /ba/ syllables in participants watching a silent film with subtitles, that is, performing also a visuo-phonological (reading) task. However, the present difference in the duration of F1 and F2 transition between the deviant /ba/ and standard /wa/ (after an initial common 5-ms transition) was 30 ms (20 ms for /ba/ vs. 50 ms for /wa/), whereas in the study of Slabu et al. (2012), it was only 15 ms (20 ms for /ba/ vs. 35 ms for /wa/) which may not have been large enough to elicit the FFR amplitude enhancement at F0 in response to deviant /ba/. In the present study, in turn, no attenuation of amplitudes at the harmonics of F0 was observed in the FFR to deviant /ba/ syllables differing more from the standard /wa/ syllables than the deviant /ba/ syllables in the study of Slabu et al. (2012).

It might be argued that fatigue or habituation of FFRs (Collet and Duclaux, 1986; Gorina-Careta et al., 2016) during the present ca. 1.5-h experiment or muscle activity and arousal (Dunlop et al., 1965) due to the visual target detection task may have affected the FFRs reported here. However, if there were such effects, they were presumably on average similar for standard and deviant syllables and therefore could not explain the differences between the FFRs elicited by these syllables. Nevertheless, the present visual phonological task was more demanding than the visual non-phonological task. Therefore, contribution of arousal differences between the tasks to the present FFR results cannot be ruled out. Still, also arousal differences between the tasks would be expected to affect similarly the FFRs to deviant and standard syllables and thus are not likely to explain the enhanced FFRs observed specifically for the deviant /ba/ syllables during the visual phonological task.

However, it should be noted that the enhancing effect of visual phonological task on the FFR elicited by a phonological deviance was observed only for one deviance type and that the effect size for the significant Task × Deviance interaction this FFR enhancement caused was rather small ($\eta_{\text{p}}^{2}$ = 0.21). Therefore, in future studies, the present results need to be replicated with a wider range of phonological deviances. Moreover, our previous studies have shown that deviant sounds eliciting the MMN and subsequent ERP components distract performance in tasks involving subsequent visual target stimuli, this distraction seen as decrease in the speed and accuracy in the visual task (e.g., Alho et al., 1997; Escera et al., 1998). Therefore, in future FFR studies, it would be of interest to clarify whether phonological deviances eliciting enhanced FFRs during a visual phonological task would distract more visual task performance than deviances not eliciting enhanced FFRs. In the present study, with independent sequences of auditorily presented syllables and visually written letters and low rates of both deviant syllables and target letters, there were too few visual target letters immediately following deviant syllables to reliably clarify this issue.

In conclusion, the present observation of enhanced FFR to deviant /ba/ syllables occurring among standard /wa/ syllables during the phonological task involving written letters suggests that active phonological processing and processing of irrelevant speech interfere. While at least up to 100 Hz FFRs get contributions not only from the subcortical ascending auditory pathway, but also from the AC (Coffey et al., 2016; Bidelman, 2018), it is not possible to resolve the origin of the present FFR enhancement. However, since cortical processing of the to-be-ignored syllables reflected by long-latency ERPs, especially the MMN elicited by deviant /ba/ syllables, was not affected by the nature of visual task performed by the participants, it is likely that the present FFR enhancement for deviant /ba/ syllables during phonological processing of written letters originated from subcortical auditory structures. The present results do not allow conclusions about the neural route through which the visual phonological processing enhanced the early processing of deviant /ba/ syllables reflected by the FFR. However, this route appears to bypass subsequent cortical processing of these phoneme deviances reflected by the MMN.

## Ethics Statement

The present study was approved by the Bioethics Committee of the University of Barcelona and conducted in accordance with the Code of Ethics of the World Medical Association (Declaration of Helsinki).

## Author Contributions

KA and CE designed the experimental protocol and data collection and analysis. KŻ programmed the experimental design and collected the data. The data were analyzed by KŻ and NG-C in supervision of KA and CE. KA, KŻ, NG-C and CE wrote the manuscript.

## Conflict of Interest Statement

The authors declare that the research was conducted in the absence of any commercial or financial relationships that could be construed as a potential conflict of interest.
